# 2-(Mesitylmethyl­sulfan­yl)pyridine *N*-oxide–18-crown-6 (2/1)

**DOI:** 10.1107/S1600536808012403

**Published:** 2008-05-03

**Authors:** B. Ravindran Durai Nayagam, Samuel Robinson Jebas, J. Jebaraj Devadasan, Dieter Schollmeyer

**Affiliations:** a Department of Chemistry, Popes College, Sawyerpuram 628 251, Tamil Nadu, India; b Department of Physics, Karunya University, Karunya Nagar, Coimbatore 641 114, India; c Department of Physics, Popes College, Sawyerpuram 628 251, Tamilnadu, India; d Institut für Organische Chemie, Universität Mainz, Duesbergweg 10-14, 55099 Mainz, Germany

## Abstract

In the title compound, 2C_15_H_17_NOS·C_12_H_24_O_6_, the asymmetric unit consists of one *N*-oxide derivative and one-half of the 18-crown-6 ether, which lies on an inversion centre. In the crown ether, the O—C—C—O torsion angles indicate a *gauche* conformation of the ethyl­eneoxy units, while the C—O—C—C torsion angles indicate planarity of these segments. In the *N*-oxide unit, the dihedral angle between the pyridine and benzene rings is 85.88 (12)°. The crystal packing is stabilized by weak C—H⋯O hydrogen bonds and C—H⋯π inter­actions.

## Related literature

For bond-length data, see: Allen *et al.* (1987 ▶). For the biological activities of *N*-oxide derivatives, see: Bovin *et al.*(1992 ▶); Katsuyuki *et al.*(1991 ▶); Leonard *et al.*(1955 ▶); Lobana & Bhatia (1989 ▶); Symons & West (1985 ▶). For related structures, see: Jebas *et al.*(2005 ▶); Ravindran Durai Nayagam *et al. *(2008 ▶).
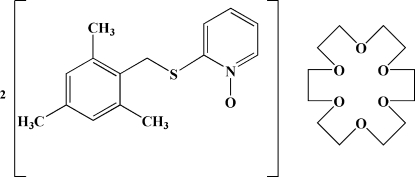


## Experimental

### 

#### Crystal data

2C_15_H_17_NOS·C_12_H_24_O_6_
*M* *_r_* = 783.02
Monoclinic, 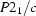

*a* = 8.050 (2) Å
*b* = 18.1903 (18) Å
*c* = 14.424 (4) Å
β = 93.475 (14)°
*V* = 2108.3 (8) Å^3^
*Z* = 2
Cu *K*α radiation
μ = 1.57 mm^−1^
*T* = 298 (2) K
0.26 × 0.22 × 0.19 mm

#### Data collection

Enraf–Nonius CAD-4 diffractometer
Absorption correction: ψ scan (North *et al.*, 1968 ▶) *T*_min_ = 0.95, *T*_max_ = 0.99 (expected range = 0.712–0.742)
3999 measured reflections
3999 independent reflections
2846 reflections with *I* > 2σ(*I*)
3 standard reflections frequency: 60 min intensity decay: 3%

#### Refinement

*R*[*F* ^2^ > 2σ(*F* ^2^)] = 0.049
*wR*(*F* ^2^) = 0.132
*S* = 1.02
3999 reflections
247 parameters
H-atom parameters constrained
Δρ_max_ = 0.21 e Å^−3^
Δρ_min_ = −0.25 e Å^−3^

### 

Data collection: *CAD-4 Software* (Enraf–Nonius, 1989 ▶); cell refinement: *CAD-4 Software*; data reduction: *CAD-4 Software*; program(s) used to solve structure: *SHELXS97* (Sheldrick, 2008 ▶); program(s) used to refine structure: *SHELXL97* (Sheldrick, 2008 ▶); molecular graphics: *SHELXTL* (Sheldrick, 2008 ▶); software used to prepare material for publication: *SHELXL97*.

## Supplementary Material

Crystal structure: contains datablocks global, I. DOI: 10.1107/S1600536808012403/ci2589sup1.cif

Structure factors: contains datablocks I. DOI: 10.1107/S1600536808012403/ci2589Isup2.hkl

Additional supplementary materials:  crystallographic information; 3D view; checkCIF report

## Figures and Tables

**Table 1:** Hydrogen-bond geometry (Å, °)

*D*—H⋯*A*	*D*—H	H⋯*A*	*D*⋯*A*	*D*—H⋯*A*
C4—H4⋯O23^i^	0.93	2.50	3.187 (3)	131
C16—H16*A*⋯O7^ii^	0.96	2.38	3.257 (4)	152
C27—H27*A*⋯*Cg*1^iii^	0.97	2.78	3.723 (4)	163
C21—H21*A*⋯*Cg*2	0.97	2.80	3.693 (3)	153
C2—H2⋯*Cg*2^iv^	0.93	2.90	3.732 (3)	150

Symmetry codes: (i) 
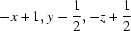
; (ii) 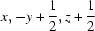; (iii) 
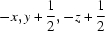
; (iv) 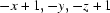. *Cg*1 and *Cg*2 are the centroids of the N6/C1–C5 and C10–C15 rings, respectively.

**Fig. 1.**
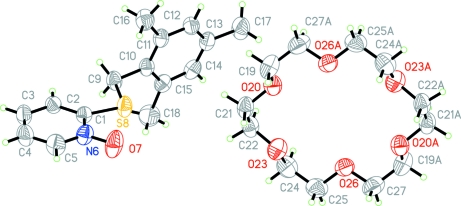
The molecular structure of the title compound, showing 50% probability displacement ellipsoids and the atomic numbering scheme. Atoms labelled with the suffix A are generated by the symmetry operation (-x, 1-y, 1-z).

**Fig. 2.**
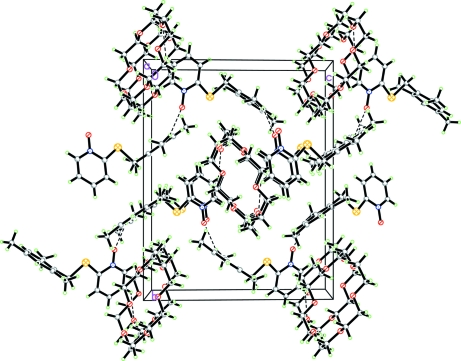
The crystal packing of the title compound, viewed along the a axis. Hydrogen bonds are shown as dashed lines.

## supplementary crystallographic information

### Comment

N-Oxides and their derivatives show a broad spectrum of biological activity, such as antifungal, antibacterial, antimicrobial and antibacterial activities (Lobana & Bhatia, 1989; Symons *et al.*,1985). These compounds are also found to be involved in DNA strand scission under physiological conditions (Katsuyuki *et al.*, 1991; Bovin *et al.*1992). Pyridine N-oxides bearing a sulfur group in position 2 display significant antimicrobial activity (Leonard *et al.*,1955). In view of the importance of N-oxides, we have previously reported the crystal structures of N-oxide derivatives (Jebas *et al.*, 2005; Ravindran Durai Nayagam *et al.*, 2008). As an extension of our work on these derivatives, we report here the crystal structure of the title compound (Fig. 1).

The asymmetric unit of the title compound consists of one mono(1-oxopyridine 2-sulfanylmethyl)mesitylene molecule and one-half of a 18-crown 6-ether molecule, the other half being inversion related. The bond lengths and angles of the N-oxide moiety agree well with those observed in other N-oxide derivatives reported earlier (Jebas *et al.*, 2005; Ravindran *et al.*, 2008). The N—O bond length is in good agreement with the mean value of 1.304 (15) Å reported in the literature for pyridine N-oxides (Allen *et al.*,1987).

The oxopyridinium and benzene rings are planar to within ±0.006 (2) Å and ±0.012 (2) Å, respectively, and they form a dihedral angle of 85.88 (12)°, indicating that both the rings are perpendicular to each other. Atom O7 deviates from the plane of the pyridinium ring by 0.006 (2) Å.

The crystal packing is consolidated by weak C—H···π interactions involving the oxopyridinium (N6/C1–C5) and benzene rings (C10–15), and C—H···O hydrogen bonds (Table 1).

### Experimental

A mixture of mono(bromomethyl)mesitylene (0.213 g, 1 mmol), 1-hydroxypyridine-2-thione sodium salt (0.1491 mmol) and 18-crown-6-ether (0.250 g) in water (30 ml) and methanol (30 ml) was heated at 333 K with stirring for 30 min. The compound formed was filtered off and dried. The compound was recrystallized from a chloroform-methanol (1:1 v/v) solution.

### Refinement

H atoms were positioned geometrically [C-H = 0.93 (aromatic), 0.96 Å (methyl) and 0.97 Å (methylene)] and refined using a riding model, with *U*_iso_(H) = 1.2-1.5*U*_eq_(C).

### Crystal data

**Table:**

2C_15_H_17_NOS·C_12_H_24_O_6_	*F*_000_ = 840
*M**_r_* = 783.02	*D*_x_ = 1.233 Mg m^−^^3^
Monoclinic, *P*2_1_/*c*	Cu *K*α radiation λ = 1.54178 Å
Hall symbol: -P 2ybc	Cell parameters from 25 reflections
*a* = 8.050 (2) Å	θ = 26–41º
*b* = 18.1903 (18) Å	µ = 1.57 mm^−^^1^
*c* = 14.424 (4) Å	*T* = 298 (2) K
β = 93.475 (14)º	Block, colourless
*V* = 2108.3 (8) Å^3^	0.26 × 0.22 × 0.19 mm
*Z* = 2	

### Data collection

**Table:**

Enraf–Nonius CAD-4 diffractometer	*R*_int_ = 0
Monochromator: graphite	θ_max_ = 70.1º
*T* = 298(2) K	θ_min_ = 3.9º
ω/2θ scans	*h* = 0→9
Absorption correction: ψ scan(North *et al.*, 1968)	*k* = 0→22
*T*_min_ = 0.95, *T*_max_ = 0.99	*l* = −17→17
3999 measured reflections	3 standard reflections
3999 independent reflections	every 60 min
2846 reflections with *I* > 2σ(*I*)	intensity decay: 3%

### Refinement

**Table:**

Refinement on *F*^2^	H-atom parameters constrained
Least-squares matrix: full	*w* = 1/[σ^2^(*F*_o_^2^) + (0.0563*P*)^2^ + 0.3138*P*] where *P* = (*F*_o_^2^ + 2*F*_c_^2^)/3
*R*[*F*^2^ > 2σ(*F*^2^)] = 0.049	(Δ/σ)_max_ < 0.001
*wR*(*F*^2^) = 0.132	Δρ_max_ = 0.21 e Å^−^^3^
*S* = 1.03	Δρ_min_ = −0.25 e Å^−^^3^
3999 reflections	Extinction correction: none
247 parameters	

### Special details

**Table:**

Geometry. All e.s.d.'s (except the e.s.d. in the dihedral angle between two l.s. planes) are estimated using the full covariance matrix. The cell e.s.d.'s are taken into account individually in the estimation of e.s.d.'s in distances, angles and torsion angles; correlations between e.s.d.'s in cell parameters are only used when they are defined by crystal symmetry. An approximate (isotropic) treatment of cell e.s.d.'s is used for estimating e.s.d.'s involving l.s. planes.

### Fractional atomic coordinates and isotropic or equivalent isotropic displacement parameters (Å^2^)

**Table:**

	*x*	*y*	*z*	*U*_iso_*/*U*_eq_	
C1	0.7135 (3)	0.08450 (12)	0.27904 (15)	0.0427 (5)	
C2	0.7560 (3)	0.01163 (13)	0.29202 (16)	0.0478 (6)	
H2	0.7162	−0.0144	0.3415	0.057*	
C3	0.8569 (3)	−0.02246 (14)	0.23183 (18)	0.0571 (7)	
H3	0.8865	−0.0715	0.2411	0.069*	
C4	0.9142 (4)	0.01540 (15)	0.1584 (2)	0.0637 (7)	
H4	0.9813	−0.0077	0.1169	0.076*	
C5	0.8717 (3)	0.08740 (16)	0.1468 (2)	0.0637 (7)	
H5	0.9106	0.1134	0.097	0.076*	
N6	0.7742 (3)	0.12176 (11)	0.20600 (15)	0.0532 (5)	
O7	0.7329 (3)	0.19062 (10)	0.19510 (16)	0.0837 (7)	
S8	0.58479 (9)	0.13969 (3)	0.34265 (5)	0.0552 (2)	
C9	0.5267 (3)	0.07413 (12)	0.43059 (17)	0.0498 (6)	
H9A	0.4587	0.0353	0.402	0.06*	
H9B	0.6256	0.0521	0.4606	0.06*	
C10	0.4303 (3)	0.11466 (12)	0.50103 (16)	0.0437 (5)	
C11	0.5156 (3)	0.15214 (12)	0.57329 (17)	0.0459 (5)	
C12	0.4274 (3)	0.19058 (13)	0.63763 (17)	0.0502 (6)	
H12	0.4853	0.2164	0.6849	0.06*	
C13	0.2556 (3)	0.19120 (13)	0.63282 (17)	0.0494 (6)	
C14	0.1730 (3)	0.15298 (13)	0.56197 (18)	0.0510 (6)	
H14	0.0573	0.1521	0.5591	0.061*	
C15	0.2554 (3)	0.11556 (13)	0.49443 (17)	0.0481 (6)	
C16	0.7035 (3)	0.15389 (16)	0.5834 (2)	0.0643 (7)	
H16A	0.7387	0.1898	0.6292	0.096*	
H16B	0.7466	0.1666	0.5249	0.096*	
H16C	0.7444	0.1063	0.6025	0.096*	
C17	0.1631 (4)	0.23629 (17)	0.7009 (2)	0.0729 (9)	
H17A	0.2288	0.2399	0.7585	0.109*	
H17B	0.059	0.213	0.7115	0.109*	
H17C	0.1428	0.2846	0.6759	0.109*	
C18	0.1546 (4)	0.07817 (17)	0.4161 (2)	0.0760 (9)	
H18A	0.1669	0.0258	0.4218	0.114*	
H18B	0.1935	0.0939	0.3577	0.114*	
H18C	0.0394	0.091	0.419	0.114*	
C19	0.3730 (4)	0.40558 (17)	0.5469 (2)	0.0732 (8)	
H19A	0.4134	0.4271	0.491	0.088*	
H19B	0.446	0.3652	0.5657	0.088*	
O20	0.2098 (2)	0.37888 (10)	0.52814 (12)	0.0592 (5)	
C21	0.2021 (4)	0.32669 (14)	0.45558 (19)	0.0601 (7)	
H21A	0.2724	0.2849	0.4727	0.072*	
H21B	0.2421	0.3485	0.3997	0.072*	
C22	0.0265 (4)	0.30186 (14)	0.43774 (19)	0.0604 (7)	
H22A	0.0223	0.2597	0.3966	0.072*	
H22B	−0.02	0.2877	0.4956	0.072*	
O23	−0.0651 (2)	0.36052 (10)	0.39661 (13)	0.0635 (5)	
C24	−0.2386 (4)	0.34718 (16)	0.3842 (2)	0.0701 (8)	
H24A	−0.2832	0.3347	0.4432	0.084*	
H24B	−0.2596	0.3064	0.3417	0.084*	
C25	−0.3196 (4)	0.41482 (17)	0.3459 (2)	0.0710 (8)	
H25A	−0.267	0.4298	0.2901	0.085*	
H25B	−0.4362	0.4051	0.3294	0.085*	
O26	−0.3054 (2)	0.47147 (10)	0.41232 (12)	0.0593 (5)	
C27	−0.3763 (4)	0.53832 (17)	0.3785 (2)	0.0751 (9)	
H27A	−0.4904	0.5298	0.3555	0.09*	
H27B	−0.3146	0.5562	0.3274	0.09*	

### Atomic displacement parameters (Å^2^)

**Table:**

	*U*^11^	*U*^22^	*U*^33^	*U*^12^	*U*^13^	*U*^23^
C1	0.0446 (12)	0.0398 (11)	0.0441 (12)	−0.0009 (10)	0.0063 (10)	0.0024 (10)
C2	0.0595 (15)	0.0396 (12)	0.0446 (13)	0.0021 (11)	0.0072 (11)	0.0024 (10)
C3	0.0721 (18)	0.0430 (13)	0.0575 (16)	0.0097 (12)	0.0141 (13)	−0.0013 (11)
C4	0.0701 (18)	0.0579 (16)	0.0655 (18)	0.0079 (14)	0.0232 (14)	−0.0043 (14)
C5	0.0668 (17)	0.0661 (17)	0.0609 (16)	0.0010 (14)	0.0273 (14)	0.0083 (14)
N6	0.0555 (12)	0.0434 (11)	0.0623 (13)	0.0026 (9)	0.0160 (10)	0.0114 (9)
O7	0.1030 (17)	0.0471 (11)	0.1058 (17)	0.0174 (11)	0.0451 (13)	0.0281 (11)
S8	0.0691 (4)	0.0367 (3)	0.0622 (4)	0.0080 (3)	0.0234 (3)	0.0054 (3)
C9	0.0619 (15)	0.0363 (12)	0.0526 (14)	0.0007 (11)	0.0147 (12)	0.0009 (10)
C10	0.0471 (13)	0.0355 (11)	0.0494 (13)	0.0004 (9)	0.0103 (10)	0.0027 (10)
C11	0.0416 (12)	0.0413 (12)	0.0554 (14)	0.0010 (10)	0.0094 (10)	0.0009 (10)
C12	0.0544 (15)	0.0465 (13)	0.0499 (14)	0.0031 (11)	0.0050 (11)	−0.0025 (11)
C13	0.0551 (15)	0.0455 (13)	0.0494 (14)	0.0106 (11)	0.0180 (11)	0.0104 (11)
C14	0.0405 (12)	0.0496 (14)	0.0639 (16)	0.0039 (11)	0.0109 (11)	0.0135 (12)
C15	0.0473 (13)	0.0403 (12)	0.0565 (14)	−0.0030 (10)	0.0026 (11)	0.0067 (11)
C16	0.0475 (15)	0.0646 (17)	0.0806 (19)	0.0003 (13)	0.0036 (13)	−0.0121 (15)
C17	0.084 (2)	0.0733 (19)	0.0652 (18)	0.0222 (16)	0.0339 (16)	0.0082 (15)
C18	0.0627 (18)	0.073 (2)	0.090 (2)	−0.0042 (15)	−0.0110 (16)	−0.0117 (18)
C19	0.0583 (17)	0.0603 (18)	0.101 (2)	0.0089 (14)	0.0081 (16)	0.0113 (17)
O20	0.0580 (11)	0.0578 (11)	0.0633 (11)	0.0006 (8)	0.0147 (9)	−0.0013 (9)
C21	0.0724 (18)	0.0469 (14)	0.0634 (17)	0.0101 (13)	0.0246 (14)	0.0093 (12)
C22	0.082 (2)	0.0395 (13)	0.0613 (16)	0.0011 (13)	0.0222 (14)	0.0054 (12)
O23	0.0668 (12)	0.0476 (10)	0.0768 (13)	−0.0066 (9)	0.0089 (10)	0.0110 (9)
C24	0.073 (2)	0.0585 (17)	0.079 (2)	−0.0173 (15)	0.0043 (16)	−0.0082 (15)
C25	0.0691 (19)	0.0725 (19)	0.0694 (19)	−0.0102 (16)	−0.0117 (15)	−0.0074 (16)
O26	0.0608 (11)	0.0588 (11)	0.0574 (11)	0.0024 (9)	−0.0048 (9)	0.0023 (9)
C27	0.0657 (19)	0.072 (2)	0.085 (2)	0.0026 (15)	−0.0183 (16)	0.0099 (17)

### Geometric parameters (Å, °)

**Table:**

C1—N6	1.368 (3)	C16—H16C	0.96
C1—C2	1.379 (3)	C17—H17A	0.96
C1—S8	1.743 (2)	C17—H17B	0.96
C2—C3	1.373 (3)	C17—H17C	0.96
C2—H2	0.93	C18—H18A	0.96
C3—C4	1.367 (4)	C18—H18B	0.96
C3—H3	0.93	C18—H18C	0.96
C4—C5	1.362 (4)	C19—O20	1.411 (3)
C4—H4	0.93	C19—C27^i^	1.482 (4)
C5—N6	1.348 (3)	C19—H19A	0.97
C5—H5	0.93	C19—H19B	0.97
N6—O7	1.303 (3)	O20—C21	1.412 (3)
S8—C9	1.823 (2)	C21—C22	1.491 (4)
C9—C10	1.508 (3)	C21—H21A	0.97
C9—H9A	0.97	C21—H21B	0.97
C9—H9B	0.97	C22—O23	1.408 (3)
C10—C11	1.392 (3)	C22—H22A	0.97
C10—C15	1.406 (3)	C22—H22B	0.97
C11—C12	1.391 (3)	O23—C24	1.418 (3)
C11—C16	1.510 (3)	C24—C25	1.483 (4)
C12—C13	1.380 (3)	C24—H24A	0.97
C12—H12	0.93	C24—H24B	0.97
C13—C14	1.374 (4)	C25—O26	1.407 (3)
C13—C17	1.509 (3)	C25—H25A	0.97
C14—C15	1.389 (3)	C25—H25B	0.97
C14—H14	0.93	O26—C27	1.417 (3)
C15—C18	1.512 (4)	C27—C19^i^	1.482 (4)
C16—H16A	0.96	C27—H27A	0.97
C16—H16B	0.96	C27—H27B	0.97
			
N6—C1—C2	118.9 (2)	C13—C17—H17B	109.5
N6—C1—S8	111.80 (17)	H17A—C17—H17B	109.5
C2—C1—S8	129.24 (18)	C13—C17—H17C	109.5
C3—C2—C1	120.0 (2)	H17A—C17—H17C	109.5
C3—C2—H2	120	H17B—C17—H17C	109.5
C1—C2—H2	120	C15—C18—H18A	109.5
C4—C3—C2	120.2 (2)	C15—C18—H18B	109.5
C4—C3—H3	119.9	H18A—C18—H18B	109.5
C2—C3—H3	119.9	C15—C18—H18C	109.5
C5—C4—C3	119.1 (2)	H18A—C18—H18C	109.5
C5—C4—H4	120.5	H18B—C18—H18C	109.5
C3—C4—H4	120.5	O20—C19—C27^i^	110.6 (2)
N6—C5—C4	121.3 (2)	O20—C19—H19A	109.5
N6—C5—H5	119.4	C27^i^—C19—H19A	109.5
C4—C5—H5	119.4	O20—C19—H19B	109.5
O7—N6—C5	121.5 (2)	C27^i^—C19—H19B	109.5
O7—N6—C1	118.0 (2)	H19A—C19—H19B	108.1
C5—N6—C1	120.5 (2)	C19—O20—C21	111.8 (2)
C1—S8—C9	100.10 (11)	O20—C21—C22	109.3 (2)
C10—C9—S8	108.26 (15)	O20—C21—H21A	109.8
C10—C9—H9A	110	C22—C21—H21A	109.8
S8—C9—H9A	110	O20—C21—H21B	109.8
C10—C9—H9B	110	C22—C21—H21B	109.8
S8—C9—H9B	110	H21A—C21—H21B	108.3
H9A—C9—H9B	108.4	O23—C22—C21	108.1 (2)
C11—C10—C15	119.5 (2)	O23—C22—H22A	110.1
C11—C10—C9	119.6 (2)	C21—C22—H22A	110.1
C15—C10—C9	120.9 (2)	O23—C22—H22B	110.1
C12—C11—C10	119.8 (2)	C21—C22—H22B	110.1
C12—C11—C16	118.3 (2)	H22A—C22—H22B	108.4
C10—C11—C16	121.9 (2)	C22—O23—C24	114.2 (2)
C13—C12—C11	121.4 (2)	O23—C24—C25	108.2 (2)
C13—C12—H12	119.3	O23—C24—H24A	110.1
C11—C12—H12	119.3	C25—C24—H24A	110.1
C14—C13—C12	118.1 (2)	O23—C24—H24B	110.1
C14—C13—C17	121.6 (2)	C25—C24—H24B	110.1
C12—C13—C17	120.2 (3)	H24A—C24—H24B	108.4
C13—C14—C15	122.7 (2)	O26—C25—C24	109.7 (2)
C13—C14—H14	118.7	O26—C25—H25A	109.7
C15—C14—H14	118.7	C24—C25—H25A	109.7
C14—C15—C10	118.5 (2)	O26—C25—H25B	109.7
C14—C15—C18	119.1 (2)	C24—C25—H25B	109.7
C10—C15—C18	122.4 (2)	H25A—C25—H25B	108.2
C11—C16—H16A	109.5	C25—O26—C27	112.3 (2)
C11—C16—H16B	109.5	O26—C27—C19^i^	110.6 (2)
H16A—C16—H16B	109.5	O26—C27—H27A	109.5
C11—C16—H16C	109.5	C19^i^—C27—H27A	109.5
H16A—C16—H16C	109.5	O26—C27—H27B	109.5
H16B—C16—H16C	109.5	C19^i^—C27—H27B	109.5
C13—C17—H17A	109.5	H27A—C27—H27B	108.1
			
N6—C1—C2—C3	−0.3 (4)	C10—C11—C12—C13	1.5 (4)
S8—C1—C2—C3	178.2 (2)	C16—C11—C12—C13	−179.8 (2)
C1—C2—C3—C4	−0.7 (4)	C11—C12—C13—C14	−0.4 (4)
C2—C3—C4—C5	1.0 (4)	C11—C12—C13—C17	−177.1 (2)
C3—C4—C5—N6	−0.2 (5)	C12—C13—C14—C15	−1.6 (4)
C4—C5—N6—O7	−179.9 (3)	C17—C13—C14—C15	175.1 (2)
C4—C5—N6—C1	−0.8 (4)	C13—C14—C15—C10	2.4 (3)
C2—C1—N6—O7	−179.8 (2)	C13—C14—C15—C18	−176.9 (2)
S8—C1—N6—O7	1.4 (3)	C11—C10—C15—C14	−1.3 (3)
C2—C1—N6—C5	1.1 (4)	C9—C10—C15—C14	179.2 (2)
S8—C1—N6—C5	−177.7 (2)	C11—C10—C15—C18	178.0 (2)
N6—C1—S8—C9	179.41 (18)	C9—C10—C15—C18	−1.6 (4)
C2—C1—S8—C9	0.8 (3)	C27^i^—C19—O20—C21	178.4 (2)
C1—S8—C9—C10	172.97 (17)	C19—O20—C21—C22	−178.8 (2)
S8—C9—C10—C11	−83.8 (2)	O20—C21—C22—O23	70.4 (3)
S8—C9—C10—C15	95.8 (2)	C21—C22—O23—C24	−174.0 (2)
C15—C10—C11—C12	−0.6 (3)	C22—O23—C24—C25	176.6 (2)
C9—C10—C11—C12	179.0 (2)	O23—C24—C25—O26	−66.6 (3)
C15—C10—C11—C16	−179.3 (2)	C24—C25—O26—C27	178.2 (3)
C9—C10—C11—C16	0.3 (3)	C25—O26—C27—C19^i^	174.7 (2)

Symmetry codes: (i) −*x*, −*y*+1, −*z*+1.

### Hydrogen-bond geometry (Å, °)

**Table:**

*D*—H···*A*	*D*—H	H···*A*	*D*···*A*	*D*—H···*A*
C4—H4···O23^ii^	0.93	2.50	3.187 (3)	131
C16—H16A···O7^iii^	0.96	2.38	3.257 (4)	152
C27—H27A···Cg1^iv^	0.97	2.78	3.723 (4)	163
C21—H21A···Cg2	0.97	2.80	3.693 (3)	153
C2—H2···Cg2^v^	0.93	2.90	3.732 (3)	150

Symmetry codes: (ii) −*x*+1, *y*−1/2, −*z*+1/2; (iii) *x*, −*y*+1/2, *z*+1/2; (iv) −*x*, *y*+1/2, −*z*+1/2;  (v) −*x*+1, −*y*, −*z*+1.
